# Nasogastric Delivery of Fecal Microbiota Transplantation for the Treatment of Fulminant *Clostridioides difficile* Infection: A Case Report

**DOI:** 10.1002/jgh3.70177

**Published:** 2025-05-13

**Authors:** Amitjeet Singh, Edward Young, Aashish Maurya, Arvind Rajagopalan

**Affiliations:** ^1^ Department of Medicine Lyell McEwin Hospital Elizabeth South Australia Australia; ^2^ Department of Gastroenterology Lyell McEwin Hospital Elizabeth South Australia Australia

**Keywords:** antibiotic‐associated diarrhea, *Clostridioides difficile* infection (CDI), fecal microbiota transplantation (FMT), nasogastric delivery, toxic megacolon

## Abstract

**Introduction:**

Clostridioides difficile infection (CDI) is a significant cause of antibiotic‐associated diarrhea with high morbidity and mortality, particularly in cases of fulminant disease. Fecal microbiota transplantation (FMT) has demonstrated efficacy in treating severe and refractory CDI, typically administered via colonoscopy. However, in cases complicated by toxic megacolon, alternative methods of FMT delivery may be necessary.

**Case Report:**

This case report describes a 46‐year‐old female with cirrhosis and fulminant CDI complicated by toxic megacolon. Due to the patient's hemodynamic instability and contraindications to endoscopic FMT delivery, a novel approach of nasogastric FMT administration was utilized. The patient received a combination of enema‐delivered and nasogastric FMT alongside standard antibiotic therapy. This approach resulted in rapid clinical improvement, with resolution of toxic megacolon, normalization of inflammatory markers, and avoidance of colectomy.

**Discussion:**

This report highlights the successful use of nasogastric FMT in a patient with fulminant CDI, offering a potential alternative delivery route when colonoscopic administration is contraindicated. To our knowledge, this is the first reported case of nasogastric FMT successfully resolving *C. difficile*‐associated toxic megacolon.

## 1 Introduction

*Clostridioides difficile* represents a challenge in healthcare settings worldwide as a leading cause of antibiotic‐associated diarrhea, with considerable morbidity and mortality [1]. There has been a concerning increase in the incidence *of C* *. difficile* infections (CDI), exacerbated by the emergence of highly virulent strains, including BI/NAP1/027 [2]. Metronidazole and oral vancomycin remain the preferred treatment options for *C. difficile* colitis, although resistant strains are increasingly prevalent [3]. There is now compelling evidence for the safety and efficacy of fecal microbiota transplantation (FMT) in severe and/or refractory *C. difficile* infection, with the most effective method being direct caecal delivery via colonoscopy [4]. However, there are often clinical challenges preventing routine colonoscopic delivery, particularly in severe cases that can be complicated by haemodynamic instability, ileus, and toxic megacolon. In this case report, we describe a patient with severe *C. difficile* infection complicated by toxic megacolon in whom colectomy was able to be avoided using nasogastric FMT delivery.

## 2 Case Report

**FIGURE 1:**
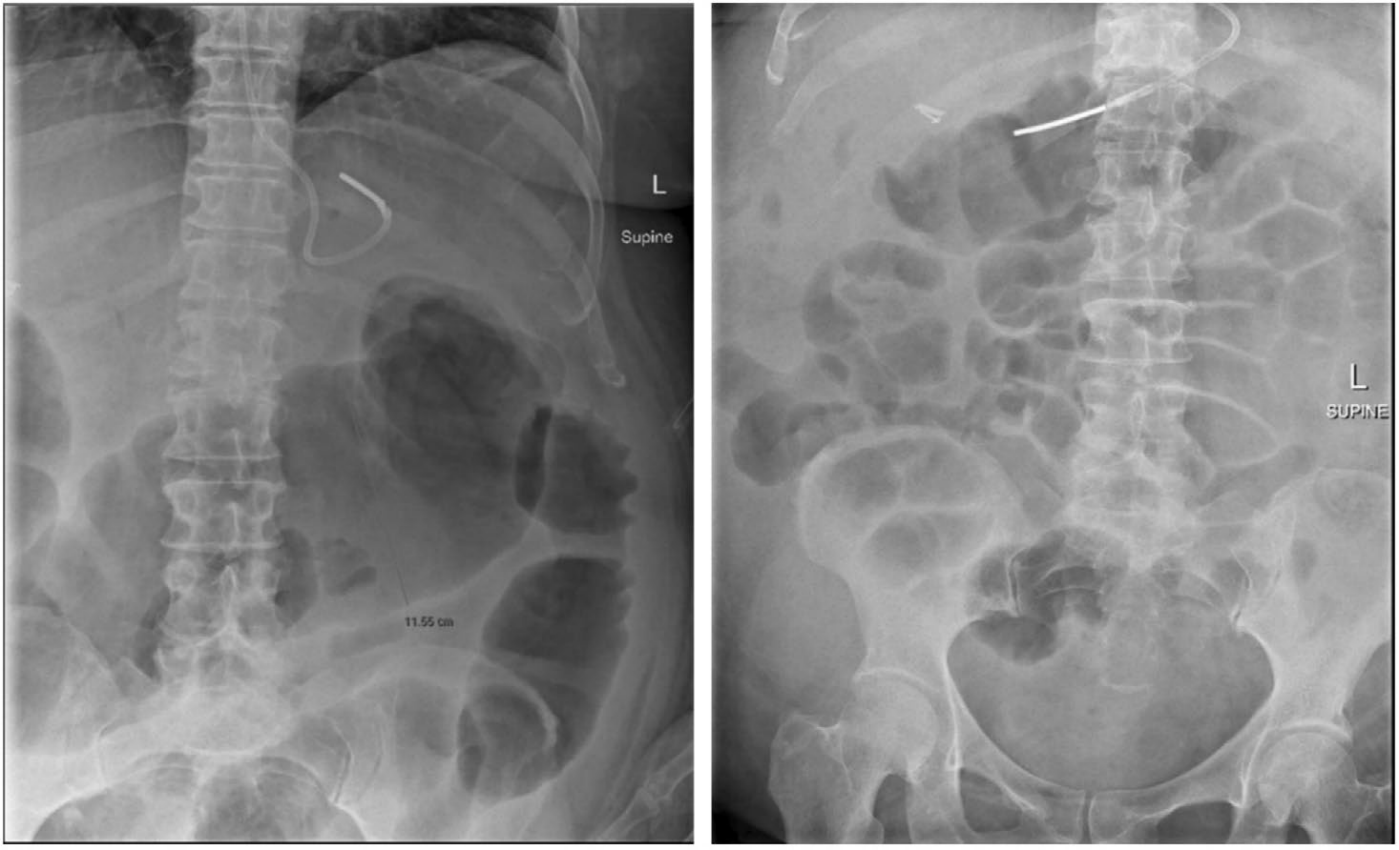
(a) Pre‐FMT abdominal x‐ray demonstrating persistently dilated transverse colon up to 12 cm. (b) Day 1 post FMT AXR demonstrating no small or large bowel dilatation. No development of any toxic dilatation. Normal amount of gas throughout large bowel.

A 46‐year‐old female with cirrhosis presented to the emergency department with haematemesis and haemodynamic instability. An emergency endoscopy demonstrated large esophageal varices with high‐risk stigmata, managed with band ligation. She was commenced on intravenous pantoprazole and ceftriaxone according to routine variceal bleeding protocols. Unfortunately, 1 week into her admission (after completing 5 days of ceftriaxone) she developed abdominal pain, diarrhea, and fevers to 38.1°C. Laboratory investigations showed a white cell count of 19 × 10^9^/L, C‐reactive protein of 143 mg/L, and albumin of 21 g/dL. Computed tomography (CT) scan of her abdomen showed colitis involving the ascending colon and splenic flexure but no colonic dilation. A stool PCR was positive for *C. difficile* toxin. Treatment was initiated with oral vancomycin 250 mg four times daily and intravenous metronidazole 500 mg three times daily. Despite escalation of vancomycin to 500 mg four times daily, the patient's symptoms worsened, with increasing abdominal pain, diarrhea, and new onset hepatic encephalopathy. Repeat abdominal x‐rays demonstrated significant colonic dilation, with a transverse colon diameter of up to 12 cm (Figure 1a).

The decision was made to proceed with FMT; however, colonoscopic delivery was not safe due to the toxic megacolon. Push enteroscopy was also deemed to be high‐risk given the recent esophageal banding, decompensated cirrhosis with ascites and encephalopathy, and ileus with resultant risk of aspiration. We administered the first 50 mL syringe of FMT solution via enema without complication. Four hours after the enema, three syringes of FMT solution were administered at two‐hour intervals via the nasogastric tube, with concurrent intravenous metoclopramide 10 mg three times a day as an anti‐emetic and prokinetic. The syringes were each delivered slowly over 10 min, ensuring no nausea or vomiting occurred.

The following day the patient had a significant improvement in abdominal pain, diarrhea, and encephalopathy. A repeat abdominal x‐ray (Figure 1b) showed the resolution of toxic megacolon with a normal amount of gas throughout the large bowel. There was significant improvement in her C‐reactive protein (67 mg/L on Day 1 post FMT and 25 mg/L on the day of discharge) and white cell count (13.2 × 10^9^/L on Day 1 post FMT and 9.6 × 10^9^/L on the day of discharge). The patient was able to be discharged home with a total 14‐day course of oral vancomycin.

## 3 Discussion

In this case, we have demonstrated the successful salvage of a *C. difficile* related toxic megacolon following the administration of FMT via a nasogastric tube, where push enteroscopy was relatively contraindicated.

Comparative studies on the efficacy of fecal transplantation based on the administration method have yielded mixed results. A systematic review found that the success rates of fecal transplantation were lower when administered via enteroscopy or nasoenteric tube (88%) compared to colonoscopy and enema (95%) [4]. Another review analyzing colonoscopic versus nasogastric methods across 12 studies revealed a higher success rate for the colonoscopic approach (93%) over nasogastric (85%), though the difference was not statistically significant [5]. Nevertheless, in severe cases, there are often contraindications to traditional methods for FMT delivery, as was the case in our patient.

There are few published articles on the use of FMT for patients with toxic megacolon, and none describe the administration of FMT via a nasogastric tube [6, 7]. Evidence has shown that FMT is effective in patients with recurrent CDI, while evidence and experience in the context of severe and fulminant *C. difficile* continue to grow [8]. To our knowledge, this is the first reported case where nasogastric delivery of FMT solution has led to the resolution of a *C. difficile‐related* toxic megacolon. FMT promotes the resolution of toxic megacolon by restoring microbial diversity, thereby re‐establishing colonization resistance against *C. difficile* and reducing toxin production. Additionally, FMT has been shown to modulate host immune responses, suppressing pro‐inflammatory cytokines and promoting epithelial repair, which is critical in severe colonic inflammation. These mechanisms collectively contribute to the reversal of colonic dysbiosis and the restoration of gut homeostasis in fulminant *C. difficile* infection [9].

There are inherent risks of aspiration with this method; however, a colectomy would have resulted in extremely high morbidity and mortality given the patient's decompensated cirrhosis. To mitigate aspiration risk during nasogastric FMT delivery, strategies such as elevating the head, using a slow infusion rate, employing prokinetic agents (metoclopramide or erythromycin), and ensuring accurate tube placement are essential [10]. This case highlights the importance of the awareness of various routes of FMT delivery in order to tailor delivery to specific patient circumstances.

## Consent

Written consent was obtained from the patient for this case report.

## Conflicts of Interest

The authors declare no conflicts of interest.
